# Development and Validation of an Early Mortality Risk Score for Older Patients Treated with Chemotherapy for Cancer

**DOI:** 10.3390/jcm10081615

**Published:** 2021-04-10

**Authors:** Jaime Feliu, Alvaro Pinto, Laura Basterretxea, Borja López-San Vicente, Irene Paredero, Elisenda Llabrés, Beatriz Jiménez-Munárriz, Maite Antonio-Rebollo, Beatriz Losada, Enrique Espinosa, Regina Gironés, Ana Belén Custodio, María del Mar Muñoz, Mariana Díaz-Almirón, Jenifer Gómez-Mediavilla, María Dolores Torregrosa, Gema Soler, Patricia Cruz, Oliver Higuera, Juan Ignacio González-Montalvo, María José Molina-Garrido

**Affiliations:** 1 Oncology Department, Hospital Universitario La Paz, IDIPAZ, Cátedra UAM-AMGEN, CIBERONC, 28046 Madrid, Spain; alvaropintomarin@gmail.com (A.P.); eespinosa00@hotmail.com (E.E.); anabcustodio@gmail.com (A.B.C.); cruz.patricia@hotmail.com (P.C.); oliverhiguera@gmail.com (O.H.); 2 Oncology Department, OSI Donostialde, Donostia Unibertsitate Ospitalea, Donostialde, Donostia, 20014 Gipuzkoa, Spain; laura.basterrecheabadiola@osakidetza.eus (L.B.); jgomez@onkologikoa.org (J.G.-M.); 3 Oncology Department, OSI Bilbao, 48013 Bilbao, Spain; borjalsv@gmail.com; 4 Oncology Department, Hospital Universitario Dr. Peset, 46017 Valencia, Spain; irene.paredero@gmail.com (I.P.); marilotorre@gmail.com (M.D.T.); 5 Oncology Department, Hospital Universitario Insular de Gran Canarias, 35016 Las Palmas, Spain; elisenda.llabres@gmail.com; 6 Oncology Department, Centro Integral Oncológico Clara Campal, 28050 Madrid, Spain; beatriz.jimenez.munarriz@gmail.com; 7 Oncohematogeriatrics Unit, Institut Català d’Oncologia, IDIBELL, Hospitalet, 08908 Barcelona, Spain; marebollo@iconcologia.net (M.A.-R.); gsoler@iconcologia.net (G.S.); 8 Oncology Department, Hospital Universitario de Fuenlabrada, 28942 Fuenlabrada, Spain; beamenta@hotmail.com; 9 Oncology Department, Hospital Universitari y Politécnic La Fé, 46026 Valencia, Spain; reginagiro@hotmail.com; 10 Oncology Department, Hospital Virgen de la Luz, 16002 Cuenca, Spain; mmmunozs@yahoo.es (M.d.M.M.); mjmolinagarrido@hotmail.com (M.J.M.-G.); 11 Biostatistics Department, Hospital La Paz, Universidad Autónoma de Madrid, 28046 Madrid, Spain; manadiaz2@gmail.com; 12 Geriatric Department, Hospital Universitario La Paz, IDIPAZ, 28046 Madrid, Spain; juanignacio.gonzalez@madrid.salud.org

**Keywords:** older patient, early death, prognostic, risk score, geriatric assessment, chemotherapy

## Abstract

*Background:* Estimation of life expectancy in older patients is relevant to select the best treatment strategy. We aimed to develop and validate a score to predict early mortality in older patients with cancer. *Patients and Methods:* A total of 749 patients over 70 years starting new chemotherapy regimens were prospectively included. A prechemotherapy assessment that included sociodemographic variables, tumor/treatment variables, and geriatric assessment variables was performed. Association between these factors and early death was examined using multivariable logistic regression. Score points were assigned to each risk factor. External validation was performed on an independent cohort. *Results:* In the training cohort, the independent predictors of 6-month mortality were metastatic stage (OR 4.8, 95% CI [2.4–9.6]), ECOG-PS 2 (OR 2.3, 95% CI [1.1–5.2]), ADL ≤ 5 (OR 1.7, 95% CI [1.1–3.5]), serum albumin levels ≤ 3.5 g/dL (OR 3.4, 95% CI [1.7–6.6]), BMI < 23 kg/m^2^ (OR 2.5, 95% CI [1.3–4.9]), and hemoglobin levels < 11 g/dL (OR 2.4, 95% CI (1.2–4.7)). With these results, we built a prognostic score. The area under the ROC curve was 0.78 (95% CI, 0.73 to 0.84), and in the validation set, it was 0.73 (95% CI: 0.67–0.79). *Conclusions:* This simple and highly accurate tool can help physicians making decisions in elderly patients with cancer who are planned to initiate chemotherapy treatment.

## 1. Introduction

Older people comprise a heterogeneous population regarding their overall health condition, functional dependence grade, comorbidities, performance status (PS), physical functional reserve, and geriatric conditions; therefore, therapeutic decisions in this population must be individualized. This represents a challenge when planning treatment. Because of that, it is interesting to identify factors that can inform us about major endpoints of clinical relevance in the decision-making process [1] such as prognostic features or treatment safety.

The prognosis of older patients with cancer may rely not only on performance status or tumor stage—as in the general population—but also on other factors such as comorbidities [1] or functional limitations [2,3]. A comprehensive geriatric assessment (CGA) evaluating these comorbidities, cognitive impairment, depression, functional status, nutritional status, and social support can be helpful for the optimization of therapeutic decisions and prognostic estimation in older patients with cancer [1,3,4,5], although the specific components of the GA that are predictive of survival are unclear [4,6]. Several scores and nomograms have been developed to estimate the risk of mortality at 1, 2, 3, and 4 years, both for the elderly population as a whole [2,7,8], and for older patients with cancer [3,9,10]. There are scores that inform about the risk of death in patients with cancer at 100 days [11] and 6 months [12], regardless of the use of antineoplastic therapy. Nevertheless, for the specific case of older people with cancer who are planned to initiate chemotherapy, even though some clinical features have been proposed as potential predictive factors for the risk of mortality [1], no scoring system is available in this regard.

We consider that estimating the risk of 6-month mortality is a relevant outcome in both early-stage and advanced-stage cancers. In the first case, it would be useful to identify those older adults at risk of early mortality after starting chemotherapy, as the long term gains in survival with adjuvant therapy may not be achieved. In the second case, a reliable prognostic estimation would allow: (1) planning therapy according to life expectancy; (2) providing information so that patients can make better decisions regarding life expectancy and priorities in this stage of the disease; (3) optimizing medical and social resources; (4) and categorizing groups of patients with similar prognosis for investigational purposes.

Nevertheless, it has been recently shown that only 30% of the estimations made by the oncologist regarding life expectancy in older adults with advanced cancer are accurate [13], so we need to develop easy and reliable tools to help us with this issue. Taking this into account, we performed a prospective, multicenter study to identify clinical, laboratory and tumor variables, and to develop a score that predicts risk of early mortality (within 6 months after the start of chemotherapy treatment) in older patients.

## 2. Materials and Methods

Our study was a multicenter study performed in 12 hospitals in Spain. We included 749 patients between February 2014 and June 2018. Main inclusion criteria were age over 70 years and histological or cytological confirmation of a nonhematological malignancy in any stage. Additional inclusion criteria were: (1) initiation of a new chemotherapy regimen (first line and beyond), (2) the ability to read Spanish (questionnaires for geriatric assessment were in Spanish), and (3) Eastern Cooperative Oncology Group performance status (ECOG-PS) 0–2. All patients provided written informed consent to participate in the study. The study was approved by the institutional review board at each participating center.

### Study Schema

Full clinical staging was performed according to routine clinical practice depending on each cancer type. Before starting chemotherapy, patients completed a baseline geriatric assessment (GA) (Supplementary Table S1). The questionnaire was delivered by a research nurse; one part was performed by the patient and another one by the health professional. The latter included the following items: ECOG PS (range 0–4) [14], comorbidities (collected using the Cumulative Illness Rating Scale and Charlson index) [15,16], frailty by the short-physical performance battery (comprising the 4 m gait-speed, standing balance, and five-repetition chair-stand test; total SPPB score ranges 0–12 points) [17,18], body mass index (BMI) and the cognitive status by the Short Portable Pfeiffer Questionnaire [19] (SPMSQ is an easily administered, validated, 10-item screen for cognitive impairment; scores 0 to 10). The patient part consisted of self-reported measures of: functional status (basic activities in daily living (ADL), scores 0–6) [20], instrumental activities in daily living (IADL) by the Lawton Index (scores 0 to 8) [21], number of falls in the last six months, medications, nutrition, psychological state (HADS score, ranging from 0 to 21 both for anxiety and depression) [22], social support and function (ranging from 5 to 25) [23,24], ability to take medications unassisted, and the Vulnerable Elders Survey-13 (VES-13, a tool to detect frailty ranging from 1 to 10) [25]. A member of the health care team assisted those who needed help with completing the questionnaires.

The following clinical variables were collected: age, gender, education, marital status, household composition, hearing, cancer subtype and stage, and selected blood tests obtained before treatment (hemoglobin, white blood cell count, platelets, basal creatinine, albumin, liver function, and creatinine clearance [26]).

All-cause mortality was captured from the hospital database and national death registry.

## 3. Statistical Analysis

The primary objective of the study was the identification of factors associated with early mortality, defined as death in the first 6 months after the beginning of a new line of chemotherapy [1]. The sample was divided into a training cohort (3 hospitals and 346 patients) and a validation cohort (9 hospitals, different from the training cohort, and 401 patients). Comparisons of baseline characteristics between the training and validation cohorts were performed using the chi-square test for categorical variables and independent *t*-tests for continuous variables.

The association between baseline characteristics (demographic, data, GA-components) and six-month mortality was analyzed using the logistic regression model. Significant variables at the 5% level and clinically relevant variables (comorbidity, BMI, age, and tumor subtype) were selected for inclusion in the multivariable model. Odds ratios (ORs) were reported with their 95% confidence intervals (CI). We performed a correlation assessment using the Spearman’s rho test as appropriate for categorical variables. Multicollinearity between variables was defined as a rho test value ≥0.50.

The two-tailed *p*-value less than 0.05 was considered statistically significant for all comparisons. Ordinal categorical variables were dichotomized according to clinically relevant cutoffs. The optimal cut-point for the laboratory values SPPB and BMI was determined using the Youden index. The cutoff usually established for frailty in the SPPB is 8, but its value for predicting mortality is not currently addressed, ranging from 4 to 8 [27,28,29]. Thus, we chose the Youden index to determine the optimal cutoff value for the SPPB. We used the bootstrapping method (1000 repetitions) to obtain a relatively unbiased estimate of the models’ performance. The amount of accounted variance was determined with the Nagelkerke correlation coefficient [R2]. Model calibration and discrimination were assessed by the Hosmer–Lameshow test and the area under the receiver operating characteristic (ROC) curve [30,31].

For the development of the score, each factor was assigned a particular score based on its β coefficient. The β coefficient for each risk factor was divided by the lowest β coefficient and rounded to the nearest whole number [32,33]. The risk score was then applied to each patient. The sample was divided into three risk strata (low, medium, and high risk of early mortality) on the basis of approximate tertiles of risk score. We compared mortality within the first 6 months among the risk groups by chi square testing. Survival by risk group was represented by Kaplan–Meier curves and *p* values were calculated using two-sided log-rank tests.

Analyses were carried out using SPSS software (version 18; SPSS, Chicago, IL, USA).

## 4. Results

A total of 749 patients completed baseline assessment. Two patients withdrew consent early, and four were treated with targeted therapies without chemotherapy and were excluded from the analysis. Baseline patient characteristics of the 743 who finally entered in the outcome analysis (342 in the training cohort and 401 in the validation cohort), show that both groups were similar except for primary cancer site, IADL, social support, creatinine clearance and gamma glutamyl transferase (GGT) levels (Table 1). Median follow-up was 27.7 months (range 0 to 30.5 months) in the training cohort, and 24.2 months (range 0–26.9) in the validation cohort. Median overall survival was not reached in any of the cohorts. A total of 76 (22%) of 342 patients in the training cohort and 75 (19%) of 401 patients in the validation cohort died in the first 6 months after chemotherapy initiation (*p* = NS). In the training cohort, 61/185 (33%) stage IV patients with stage IV disease and 15/156 (10%) of those with localized disease died in the first 6 months, whereas in the validation cohort, this happened in 61/221 (28%) and 14/180 (8%), respectively (*p* = NS). Causes for early death in the training and validation cohort were significantly different: toxicity 4 (5%) vs. 3 (1%), comorbidities 29 (38%) vs. 13 (17%) and disease progression 43 (57%) vs. 59 (79%); (*p* < 0.05).

## 5. Predictive Variables Associated with the Risk of Death in the First 6 Months

**Figure 1:**
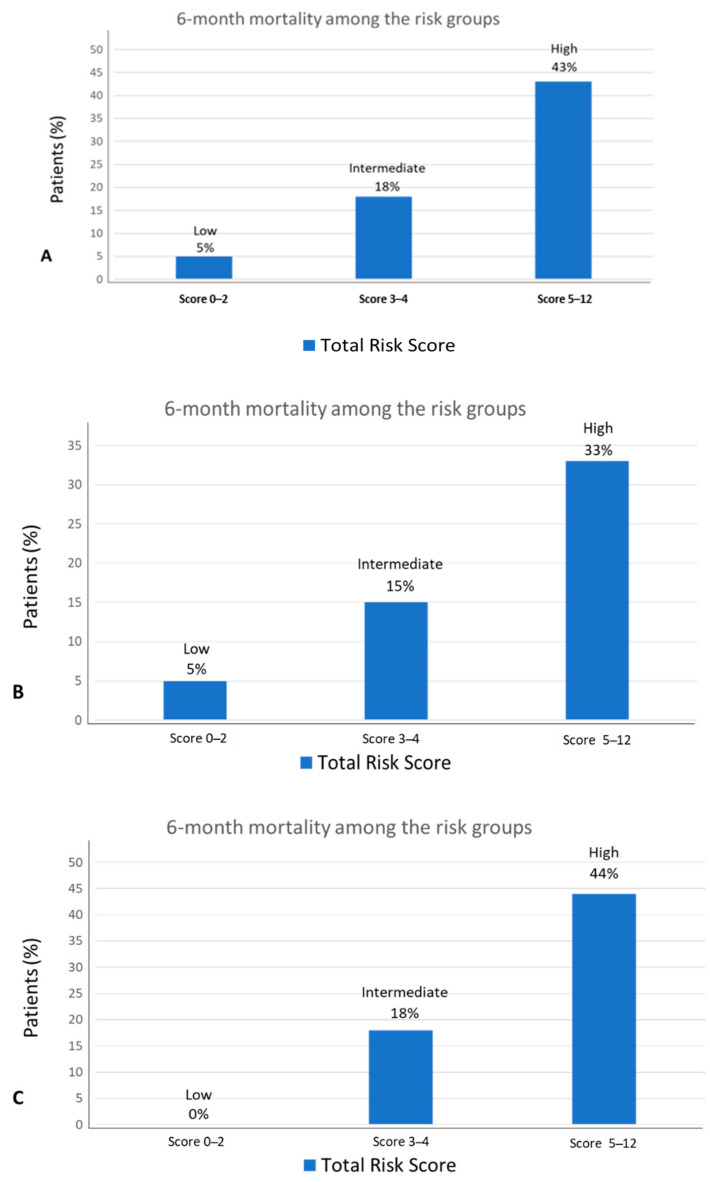
Ability of risk score to predict 6-month mortality (**A**) in the development cohort, (**B**) in the early stage, and (**C**) metastatic stage.

**Figure 2:**
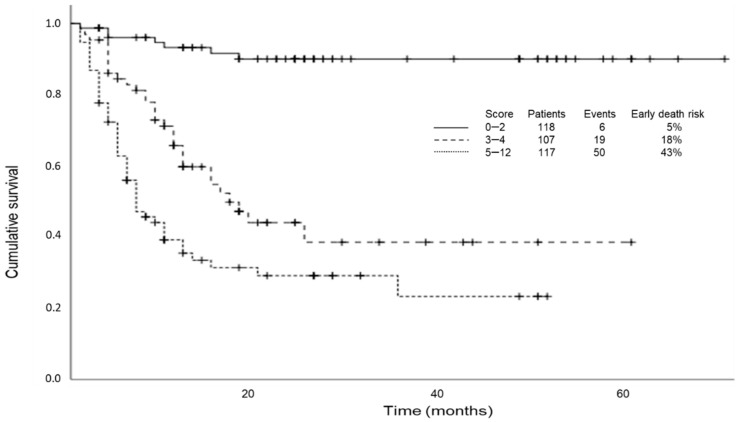
Kaplan–Meier survival curves by risk score groups.

In the univariable survival analysis from the training cohort, we identified 14 factors that were associated with early death (within 6 months) (Table 2). The risk factors associated with 6-month mortality in univariable analysis (*p* < 0.05) and variables deemed to be of clinical significance (i.e., comorbidity, age, and tumor subtype) were included in the multivariable model. In the multivariable model, only six independent variables were directly associated with survival time: metastatic stage, ECOG PS, ADL, serum albumin levels, BMI, and hemoglobin levels (Table 3). All of the cancer diagnosis groups had similar distributions for these prognostic variables. The risk score was applied to each patient, and patients were classified into three risk score groups using approximate tertiles: low risk (0–2 points: 5% 6-month mortality rate), intermediate risk (3 to 4 points: 18% 6-month mortality rate), and high risk (5 to 12 points: 43% 6-month mortality rate). The proportion of patients classified as low, intermediate, or high risk were 35%, 31%, and 34% respectively (Figure 1). There was a significant difference in the mortality within the first 6 months among the risk groups (*p* < 0.001). Median overall survival was also significantly different between the three risk groups: low risk, not reached; intermediate, 17 months (95% CI: 10.8–23.1) and high risk, 7 months (95% CI: 4.0–9.9) (*p* < 0.000) (Figure 2).

## 6. Model Prediction of Early Mortality

The area under receiver operation characteristic (ROC) curve for the training cohort was 0.785 (95% CI: 0.73–0.84). (Supplementary Figure S1). The average ROC after bootstrap resampling decreased marginally to 0.77 (95% CI: 0.72–0.83), suggesting that the model is internally valid. Exploratory analyses were performed to calculate the ROC of the model using the total risk score according to the stage: localized (0.79) and disseminated (0.72), and for each tumor type: GI (0.81), GU (0.88), breast (0.84), lung (0.70), and other (0.71). The performance of the proposed score in localized and advanced disease is shown in Figure 1. Causes of death in the first 6 months outlined by tumor stage are shown in Supplementary Table S2. Calibration of the final model was assessed using the Hosmer–Lemeshow goodness of fit test. A *p*-value of 0.79 (95% CI, 0.74 to 0.85) suggests that the model is accurate. The model retained adequate discrimination in the external validation cohort with a ROC of 0.73 (95% CI: 0.67–0.79) (Supplementary Figure S1). The performance of the score in the validation set is shown in Supplementary Figure S2.

## 7. Discussion

Older patients have the same willingness to receive chemotherapy than other younger groups [34], but they demand a longer survival benefit and less toxicity [35]. Because of that, accurate estimation of life expectancy is critical to plan the treatment strategy.

We have developed a tool that predicts death within 6 months of starting chemotherapy using basic clinical and analytical information. Multivariable analysis results suggest that tumor stage, ECOG performance status, ADL, serum albumin levels, BMI, and hemoglobin levels are independent risk factors of early death.

To our knowledge, this is the first prognostic score to address the risk of early death in older patients with cancer who are going to initiate chemotherapy. The variables included in our score had been identified as prognostic factors in previous studies. Variables were related with the geriatric assessment (nutritional status and functional capabilities), the tumor itself, and with some analytical features (hemoglobin and albumin). In a general geriatric population and also in older cancer patients, nutritional status is considered a key prognostic factor [36,37], although its evaluation differs between studies [1,3,11]. In our series, nutrition-related features with independent prognostic value were BMI and albumin levels, as seen in some other studies [3,9,11,38]. Although serum albumin level is used to evaluate protein reserves, this parameter also has been related to survival time in advanced cancer patients [39,40], overall survival in older cancer patients [3,38] and risk of early death [1,11].

Another essential part of geriatric assessment is functional status. In multivariate analysis, both ECOG PS and daily life activities limitations had prognostic implications. Although both variables evaluate functional status, they seem to offer complementary information [41]. Some other studies have also proved the value of ECOG PS [1,3,11] and ADL evaluation [9,42] to predict the risk of early mortality in older adults with cancer. Other authors have also described the relationship between tumor stage and risk of early death [1,3,9,10,12]. Similarly, anemia has also been identified as a risk factor for early death in previous studies of elderly patients with cancer [43]. Although anemia in a patient with cancer is usually multifactorial, the most common cause in this population is inflammation–chronic disease [44]. Cancer itself causes a systemic proinflammatory status that inhibits hematopoiesis [45] and favors cancer progression, affecting the prognosis of the malignant disease.

The six variables of our risk score are easy to obtain, and usually are part of the information already available in daily practice. This leads to almost no significant time-consuming efforts to apply our score. We believe that its simplicity and availability may improve its use in clinical practice.

Other scores used to predict survival in older adults with cancer have included measures such as gait speed [11,12], or a mini nutritional assessment [10,12,36], which are less easily implemented into everyday practice. Currently available scores to predict early mortality in older adults with cancer (at 100 days [11] and at 6 months [12]) were developed in heterogeneous populations regarding types of treatment, including patients both on active treatment and only on palliative measures. Our study included only patients planned to begin chemotherapy, making it more useful to oncologists seeing such patients than scores with more heterogeneous populations.

The overall 6-month mortality rate in our series was 20%, higher than previously reported (16–17%) [1,12]. Nevertheless, these variations can be possibly attributed to different patients’ characteristics, mainly the rate of patients with stage IV disease, type of tumor, and the inclusion of patients in any line of therapy (first line and beyond).

Apart from the simplicity of the score, its main strength is that is has been externally validated with patients from different cancer centers and with a variety of clinical features so that the results could be generalized. However, the score should be explored in other countries and should be validated in everyday practice. Regarding its limitations, most of our patients had digestive cancer, whereas other primary locations were relative rare or absent, as it was the case with hematological malignancies and brain tumors, so the performance of the score in patients with tumor types not represented in this study cannot be inferred. Although AUC was good, some variation remains unexplained: the inclusion of additional variables, such as “grip strength” or “timed get up and go” could have improved prognostic accuracy.

We think that this tool may help in the decision-making process. For instance, if a patient who was considering adjuvant chemotherapy for early stage colon cancer fell into the high-risk group, with a risk of dying within 6 months of 43%, we should avoid adjuvant therapy, as there is little chance to obtain a benefit from an otherwise toxic treatment. In a similar way, if a patient planned to initiate a second-line chemotherapy for advanced gastric cancer fell within the high-risk group, the possibilities of having any survival benefit are low, and maybe we should focus on symptom control.

In conclusion, we have developed a validated score for predicting the probability of early death for older patients with cancer that are planned to initiate chemotherapy. This score can aid in estimating 6-month mortality and, therefore, in making decisions to improve the care of our patients. This tool may classify patients into homogeneous prognostic groups in order to better stratify patients’ characteristics for clinical research.

## Figures and Tables

**Table 1:** Characteristics of patients in the training set and the validation set.

	Training Set No. (%)	Validation Set No. (%)	*p* Value
No. of patients	342	401	
No. of early deaths (≤6 months)	76 (22)	75 (19)	0.10
Sex			0.81
Female	140 (41)	157 (39)	
Male	202 (59)	244 (61)	
Age (Median and range)	78 (70–92)	77 (70–89)	0.74
Primary cancer sites			0.001
Gastrointestinal	205 (60)	193 (48)	
Urinary organs	41 (12)	27 (7)	
Lung	31 (9)	85 (22)	
Pancreatic cancer	24 (7)	12 (3)	
Breast	15 (4)	22 (5)	
Female reproductive	12 (4)	19 (5)	
Others	14 (4)	43 (11)	
Stage IV	185 (54)	221 (55)	0.78
ECOG performance status 2	44 (13)	61 (15)	0.19
Weight change in the last 6 months ≥ 10%	42 (12)	67 (9)	0.09
Activity of daily living ≤ 5	74 (22)	88 (20)	0.61
Instrumental activity of daily living ≤ 7	203 (59)	197 (49)	0.005
Body mass index (Mean and SD)	26.1 (4.1)	25.6 (6.1)	0.2
Charlson score ≥ 2	125 (37)	129 (32)	0.20
Number of falls in the past 6 months ≥ 1	69 (20)	57 (14)	0.16
SPPB < 6	63 (18)	64 (16)	0.19
Pfeiffer test ≥ 3 errors	45 (13)	40 (10)	0.17
Hospital Anxiety Scale ≥ 8	72 (21)	76 (19)	0.47
Hospital Depression Scale ≥ 8	79 (23)	80 (20)	0.29
MOS Social Support Survey ≤ 15	40 (12)	24 (6)	0.005
VES-13 ≥ 3	184 (54)	195 (49)	0.15
Baseline laboratory values (Mean and SD)			
Hemoglobin (g/dL)	12.5 (1.6)	12.6 (1.6)	0.22
Albumin (g/dL)	3.9 (0.4)	3.8 (0.5)	0.29
Creatinine clearance (mL/min)	47 (22)	69 (20)	0.01
Gamma glutamyl transferase (IU/L)	108 (191)	82 (170)	0.002

Abbreviations: No, number; ECOG PS, Eastern Cooperative Oncology Group performance status; SPPB, short-physical performance battery; MOS, medical outcomes study; VES-13, Vulnerable Elders Survey-13; SD, standard deviation.

**Table 2:** Factors associated with early mortality on univariable analysis.

Variable	Patients	Died (*n* = 76)	Alive (*n* = 266)	OR (95% CI)	*p*-Value
	*n* (%)	*n* (%)	*n* (%)		
ECOG PS				2.28 (1.18–4.40)	0.013
0–1	298 (87)	58 (19)	240 (81)		
2	44 (13)	18 (41)	26 (59)		
ADL				1.76 (1.01–3.09)	0.046
6	268 (78)	51 (19)	217 (81)		
≤5	74 (22)	25 (34)	49 (65)		
IADL				1.67 (1.01–2.76)	0.042
8	139 (41)	26 (19)	113 (81)		
≤7	203 (59)	50 (65)	153 (75)		
SPPB				1.99 (1.11–3.58)	0.021
>7	279 (82)	53 (19)	226 (66)		
≤6	63 (18)	23 (37)	40 (63)		
Tumour site				1.53 (1.06–2.19)	0.021
Lung	31 (9)	13 (42)	18 (58)		
Other	311 (91)	63 (20)	248 (80)		
VES 13				1.59 (1.01–2.61)	0.05
0–2	168 (49)	28 (17)	140 (83)		
>3	174 (51)	48 (28)	126 (72)		
Stage				4.71 (2.62–8.46)	<0.001
I-III	157 (46)	15 (10)	142 (90)		
IV	185 (54)	61 (33)	124 (67)		
Neutrophils (×10^3^/µL)					
≤8	195 (57)	27 (14)	168 (86)	1.48 (1.15–1.90)	0.002
>8	147 (43)	49 (33)	98 (67)		
GGT (IU/L)				1.53 (1.17–2.04)	0.002
≤130	261 (76)	45 (17)	216 (83)		
>130	81 (24)	31 (38)	50 (62)		
Alkaline Phosphatase (IU/L)				2.81 (1.59–4.97)	<0.001
≤150	276 (81)	49 (18)	227 (82)		
>150	66 (19)	27 (41)	39 (59)		
Albumin (g/dL)				4.74 (2.64–8.51)	<0.001
≥3.5	281 (82)	44 (16)	237 (84)		
<3.5	61 (18)	32 (52)	29 (48)		
Hemoglobin (g/dL)				2.51 (1.43–4.40)	0.001
≥11	272 (80)	48 (18)	224 (82)		
<11	70 (20)	28 (40)	42 (60)		
Weight loss %				2.06 (1.04–4.06)	0.037
<10%	301 (88)	60 (20)	241 (80)		
≥10%	41 (12)	16 (39)	25 (61)		
BMI (kg/m^2^)				2.14 (1.20–3.81)	0.01
≥23	271 (79)	50 (18)	221 (82)		
<23	71 (21)	26 (34)	45 (17)		

Abbreviations: ECOG PS, Eastern Cooperative Oncology Group performance; ADL, activities of daily living; IADL: instrumental activities of daily living; SPPB, short-physical performance battery; VES-13, Vulnerable Elders Survey-13; GGT, gamma glutamil transferase; Alkaline Ph, alkaline phosphatase; BMI, body mass index. IU, international unit.

**Table 3:** Variables significantly associated with early mortality on multivariable analysis.

Variable	β	SE	*p* ^†^	OR (95% CI)	Score
BMI (<23 kg/m^2^)	0.912	0.343	0.008	2.489 (1.271–4.875)	2
Stage IV	1.573	0.353	0.000	4.820 (2.414–9.626)	3
Albumin (≤3.5 g/dL)	1.210	0.347	0.000	3.353 (1.698–6.622)	2
Hemoglobin (<11 g/dL)	0.868	0.344	0.012	2.383 (1.213–4.681)	2
ADL (≤5)	0.565	0.351	0.041	1.760 (1.085–3.501)	1
ECOG PS 2	0.868	0.402	0.031	2.382 (1.084–5.232)	2

Abbreviations: OR, odds ratio; CI, confidence interval; BMI, body mass index; ADL, activity of daily living; ECOG, Eastern Cooperative Oncology Group; ^†^ *p*-values were calculated using a two-sided Wald test for multivariable analyses.

## Data Availability

De-identified individual data might be made available following publication by reasonable request to the corresponding author.

## Supplementary Materials

The following are available online at https://www.mdpi.com/article/10.3390/jcm10081615/s1, Table S1: Summary of Comprehensive Geriatric Assessment Domains and Elements; Figure S1: Receiver operating characteristic (ROC) analyses to assess the capacity of the prognostic score to predict death at 6 months among the 342 patients in the training set (A) and in the 401 patients in the validation set (B); Figure S2: Ability of risk score to predict 6-month mortality in validation cohort; Table S2: Reasons for early death in older patients with cancer treated with chemotherapy.
