# Supplementary material for: Development and Validation of an Early Mortality Risk Score for Older Patients Treated with Chemotherapy for Cancer
Source: J Clin Med. 2021 Apr 10;10(8):1615. doi: 10.3390/jcm10081615 (PMC8070509; doi:10.3390/jcm10081615)
Supplement: Supplementary file 1 [file jcm-10-01615-s001.pdf]

Journal of
Clinical Medicine

Suppelementary Material

Supplementary Table 1. Summary of Comprehensive Geriatric Assessment Domains and Elements

Domain	Elements of Assessment
Functional status	ECOG performance status<br>Activities of daily living [1]<br>Instrumental activities of daily livig [2]<br>Physical performance test SPPB [3]<br>Nº of falls in the last 6 months
Comorbidity	Cumulative Illness Rating Scale for Geriatrics (CIRS-G score) [4]<br>Charlson index [5]
Psychological status	Hospital Anxiety Scale [6]
Cognitive status	Pfeiffer test [7]
Social support	MOS Social Support Survey [8]
Nutritional status	Body mass index<br>Percent unintentional weight lost in the last 6 months

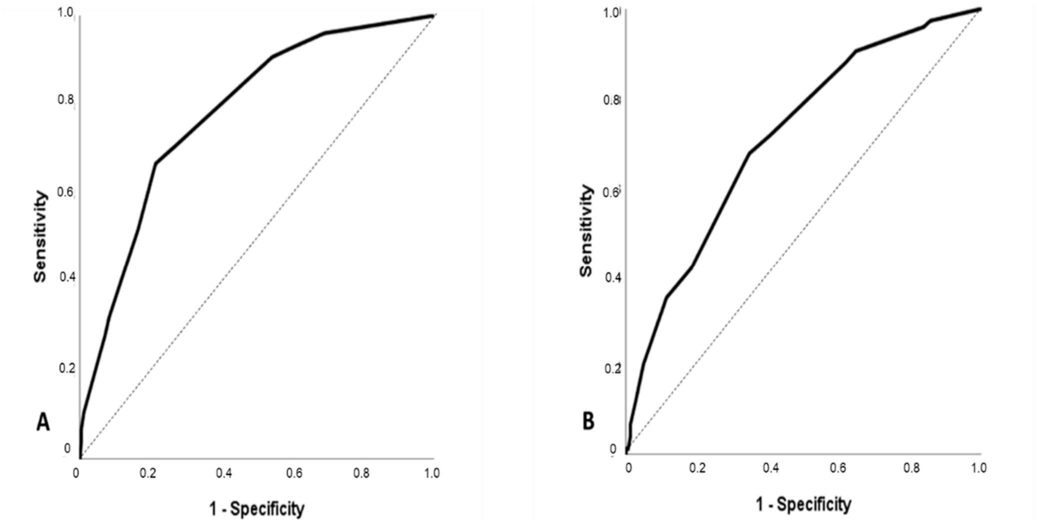

Supplementary Figure S1. Receiver operating characteristic (ROC) analyses to assess the capacity of the prognostic score to predict death at 6 months among the 342 patients in the training set (A) and in the 401 patients in the validation set (B).

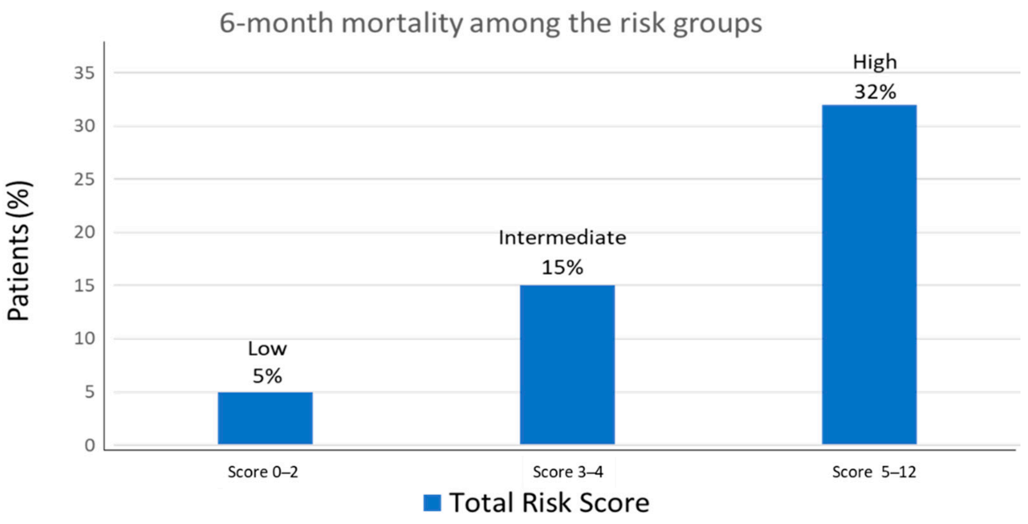

Supplementary Figure S2. Ability of risk score to predict 6-month mortality in validation cohort.

Supplementary Table S2. Reasons for early death in older patients with cancer treated with chemotherapy.

Reported cause	Stage I–III	Stage IV	Total
	***n* = 15**	***n* = 61**	***n* = 76**
Chemotherapy toxicity	3 (20%)	1 (2%)	4 (5%)
Comorbidities	8 (53%)	21 (34%)	29 (38%)
Disease progression	4 (27%)	39 (64%)	43 (57%)

Reference

1. Katz, S.; Ford, A.B.; Moskowitz, R.W.; Jackson, B.A.; Jaffe, M.W. Studies of illness in the aged: The index of ADL—A standardized measure of biological and psychosocial function. *JAMA* **1963**, *185*, 914–919.
2. Lawton, M.P.; Brody, E.M. Assessment of older people: Self-maintaining and instrumental activities of daily living. *Gerontologist* **1969**, *9*, 179–186.
3. Guralnik, J.M.; Simonsick, E.M.; Ferrucci, L.; Glynn, R.J.; Berkman, L.F.; Blazer, D.G.; Wallace, R.B. A short physical performance battery assessing lower extremity function: Association with self-reported disability and prediction of mortality and nursing home admission. *J. Gerontol.* **1994**, *49*, 85–94.
4. Linn, B.S.; Linn, M.W.; Gurel, L. Cumulative illness rating scale. *J. Am. Geriatr. Soc.* **1968**, *16*, 622–626.
5. Charlson, M.E.; Pompei, P.; Ales, K.L.; MacKenzie, C.R. A new method of classifying prognostic comorbidity in longitudinal studies: Development and validation. *J. Chronic Dis.* **1987**, *40*, 373–383.
6. Zigmod, A.S.; Snaith, R.P. The hospital anxiety and depression scale. *Acta Psychiatr. Scan.* **1983**, *67*, 361–370.
7. Pfeiffer, E.A. A short portable mental status questionnaire for the assessment of organic brain deficits in elderly patients. *J. Am. Geriatr. Soc.* **1975**, *22*, 433.
8. Ren, X.S.; Skinner, K.; Lee, A.; Kazis, L. Social support, social selection and self-assessed health status: Results from the veterans health study in the United States. *Soc. Sci. Med.* **1999**, *48*, 1721–1734.
